# THE ROLE OF SOCIAL MEDIA COMMUNICATION IN THE ASSOCIATION BETWEEN WIDOWHOOD AND DEPRESSIVE SYMPTOMS

**DOI:** 10.1093/geroni/igad104.2155

**Published:** 2023-12-21

**Authors:** Nahyun Kim, Youjin Kim, Hyeonji Cho, Kyungmin Kim

**Affiliations:** Seoul National University, Seoul, Republic of Korea; Seoul National University, Seoul, Republic of Korea; Seoul National University, Seoul, Republic of Korea; Seoul National University, Seoul, Republic of Korea

## Abstract

Losing a spouse is one of most painful experiences in adulthood; widowed adults often suffer from psychological difficulties. Scholars have examined how social support from close relationships such as family members and friends plays a protective role in the context of spousal loss. Given that the use of mobile technologies and internet became an important way to engage with social relationships, it is crucial to investigate the role of social media communication in the bereavement process. This study examined the moderating effects of social media communication in the association between widowhood and depressive symptoms, comparing different types of social media communication—chatting/messaging (e.g., iMessage), social media (e.g., Facebook), video-sharing (e.g., YouTube), and online community platforms (e.g., forums). From the 2021 Korea Social Integration Survey, we analyzed a sample of 5,238 respondents (aged 40+) who were ever-married. Our regression models showed that widowed respondents (9.79%) were likely to have more depressive symptoms (B = 0.19, p < .001) than non-widow(er)s; all types of social media communication were associated with fewer depressive symptoms, regardless of widowhood. We also found that two types of social media communication moderated the association between widowhood and depressive symptoms; such that widowed respondents who used social media (B = -0.08. p =.002) and online community platforms (B = -0.05. p = .038) more frequently had fewer depressive symptoms, compared to their counterparts. Our findings highlight the importance of examining social engagement via information and communication technologies and the potential mechanism contributing to widowed adults’ well-being.

